# Autophagy in Chronic Kidney Diseases

**DOI:** 10.3390/cells8010061

**Published:** 2019-01-16

**Authors:** Tien-An Lin, Victor Chien-Chia Wu, Chao-Yung Wang

**Affiliations:** 1 Department of General Surgery, Chang Gung Memorial Hospital, Taoyuan City 333, Taiwan; doroz1119@gmail.com; 2 Department of Cardiology, Chang Gung Memorial Hospital and Chang Gung University College of Medicine, Taoyuan City 333, Taiwan; victorcwu@hotmail.com; 3 Institute of Cellular and System Medicine, National Health Research Institutes, Zhunan 350, Taiwan

**Keywords:** autophagy, kidney diseases, oxidative stress, inflammation, mitochondria

## Abstract

Autophagy is a cellular recycling process involving self-degradation and reconstruction of damaged organelles and proteins. Current evidence suggests that autophagy is critical in kidney physiology and homeostasis. In clinical studies, autophagy activations and inhibitions are linked to acute kidney injuries, chronic kidney diseases, diabetic nephropathies, and polycystic kidney diseases. Oxidative stress, inflammation, and mitochondrial dysfunction, which are implicated as important mechanisms underlying many kidney diseases, modulate the autophagy activation and inhibition and lead to cellular recycling dysfunction. Abnormal autophagy function can induce loss of podocytes, damage proximal tubular cells, and glomerulosclerosis. After acute kidney injuries, activated autophagy protects tubular cells from apoptosis and enhances cellular regeneration. Patients with chronic kidney diseases have impaired autophagy that cannot be reversed by hemodialysis. Multiple nephrotoxic medications also alter the autophagy signaling, by which the mechanistic insights of the drugs are revealed, thus providing the unique opportunity to manage the nephrotoxicity of these drugs. In this review, we summarize the current concepts of autophagy and its molecular aspects in different kidney cells pathophysiology. We also discuss the current evidence of autophagy in acute kidney injury, chronic kidney disease, toxic effects of drugs, and aging kidneys. In addition, we examine therapeutic possibilities targeting the autophagy system in kidney diseases.

## 1. Introduction

Autophagy is a dynamic cellular balancing mechanism for energy and resource. The word autophagy is derived from the Greek word, where *auto* means “self” and *phagy* means “eating”. This “self-eating” process helps cells recycle their endogenous materials and build essential macromolecules to maintain cellular homeostasis and reutilize energy [1,2]. Autophagy was initially referred to the catabolic process that could provide nutrition and energy to cells during starvation. Recently, more evidence has shown that autophagy plays a critical role in synthesis and degradation and has complex cross-talks to apoptosis and cell cycle regulations [3]. Thus, autophagy acts as a protective mechanism in living organisms and can interfere in pathogenesis [4,5].

In 1963, Christian de Duve first described the autophagy as the degradation process that occurred after cytoplasmic materials were delivered to the lysosome. In the 1990′s the autophagy research bloomed after the team of Dr. Yoshinori Ohsumi identified the important genes related to the autophagy-defective mutants in yeasts called the autophagy-related gene (Atg) and its related protein [6]. Soon after, the research advances that focused on the genetic aspects and several Atg proteins were discovered [4,5], providing a better understanding of the function and mechanisms of autophagy. Recently, autophagy has been widely implicated not only in yeasts and animal models but also in human pathophysiological processes [5,7]. In 2016, Yoshinori Ohsumi was awarded the Nobel Prize in Physiology or Medicine in recognition for his work on laying the foundation for a better understanding of the ability of cells to manage starvation, stress, and diseases [4].

**Figure 1:**
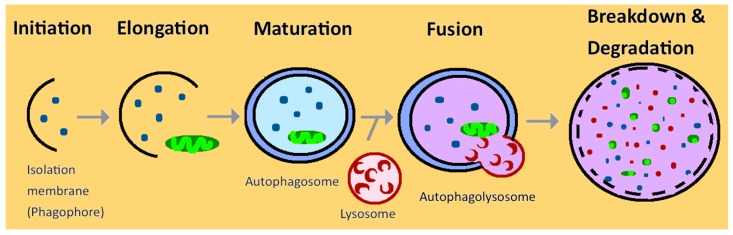
Schematic overview of the normal autophagy function in the kidney. Multiple steps in autophagy are modulated in kidney diseases, including autophagy initiation, elongation, maturation, fusion, and final degradation and recycling.

Autophagy works through intracellular lysosomal degradation and recycling, and in turn, helps to maintain cellular integrity more efficiently by regenerating metabolic precursors and clearing subcellular debris [5]. Autophagy is a series of catabolic processes, starting with a small membrane phagophore in the cytoplasm and elongating to form a cup-shaped structure. It then matures and becomes a double-membrane structure called autophagosome that engulfs the damaged components. The autophagosome then fuses with lysosomes to form autophagolysosome [4,8] (Figure 1). After forming the autophagolysosome, the mTOR (mammalian target of rapamycin) signaling is activated to degenerate the intercellular components and transport back to the cytoplasm to reuse the macromolecule [9].

Defective autophagy signaling is now found in multiple diseases, such as autoimmune diseases, infectious diseases, metabolic diseases, muscular disorders, neurodegenerative diseases, cardiovascular and pulmonary diseases [5,7,10]. Many pathophysiologic mechanisms, including ischemic, toxic, infection, oxidative stress, circadian rhythm, and aging, are also confirmed to have close interactions with autophagy. In certain stressed situations, selective autophagy occurs in order to remove toxic materials within cells and organs [1,11]. Currently, only a few studies review the relationship between kidney diseases and autophagy. Therefore, our aim is to summarize the recent advances in understanding the role of autophagy in acute and chronic kidney disease patients [12].

## 2. Autophagy in Normal Kidney

Previous studies from human and animals provide the evidence that autophagy has a great impact on the maintenance of renal functions and homeostasis [13]. However, autophagy is nonessential for embryonic renal development. The embryonic *Atg5* knockout mice show no significant impairment of glomerular development, no change in podocyte maturation, normal tubular function, and normal nephrons development [3,14]. In terms of kidney physiology in adult animals, the autophagy affects different renal cell types and helps maintain kidney pathophysiology and homeostasis [13].

### 2.1. Autophagy in Glomerular Mesangial Cells

Glomerular mesangial cells are located in the mesangium of the glomerulus, in the centrilobular region of the kidneys. Mesangial cells are specialized pericytes with contractile activities. They regulate glomerular filtration and act as primary producers of the extracellular matrix that constitutes the mesangium, thus playing a vital role in maintaining mesangial matrix homeostasis [3]. Autophagy plays dual roles in modulating mesangial cell survival. After mesangial cells are exposed to stress, autophagy is activated and directed to type II programmed cell death. At the same time, autophagy can also serve as a protective role through transforming growth factor-β1 (TGF-β1) activation and inhibit mesangial cells from apoptosis and necrosis [3].

The advanced glycation end-products (AGEs) induced mesangial cells injury is one of the leading causes of glomerular dysfunction in diabetic nephropathy [15]. Autophagy could serve as a protective manner through increased LC3 cleavage (LC3-II/LC3-I ratio), enhanced Atg5 protein expression, and decreased p62 level in mesangial cells in dose- and time-dependent manners after exposed to AGEs. Also, inhibition of Atg5 expression could aggravate AGEs related mesangial cells injury [16]. This indicates that autophagy may protect mesangial cells from apoptosis. Previous studies suggest that the AGEs could induce autophagy through a RAGE/PI3K/AKT/mTOR signaling pathway in cardiomyocytes [17].

**Figure 2:**
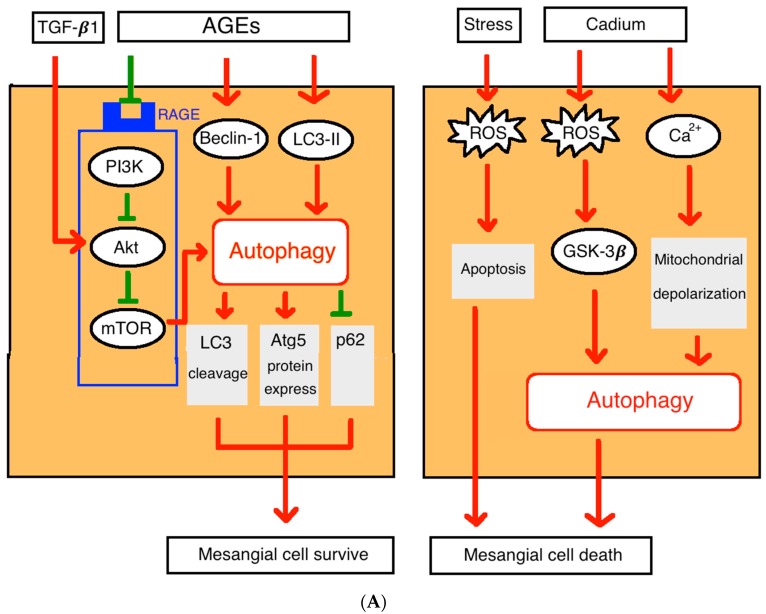

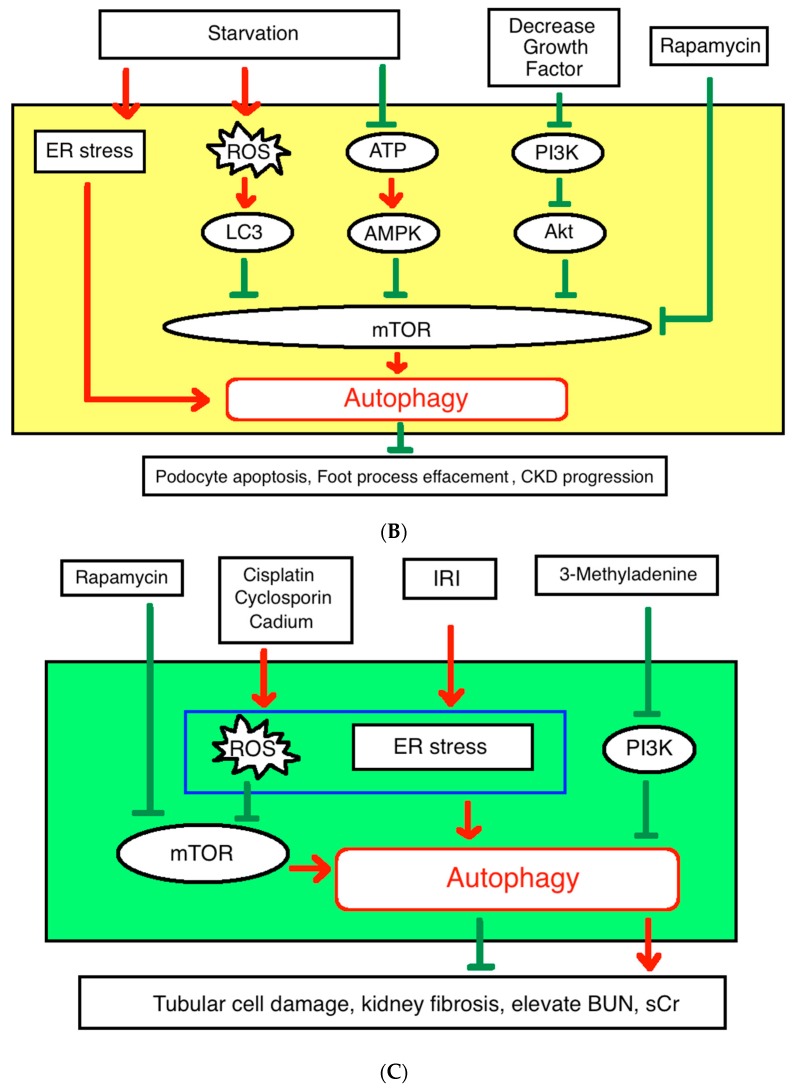
(**A**). Autophagy in glomerular mesangial cells. AGEs: advanced glycation end-products; Akt: also stand for PKB (Protein kinase B); GSK-3β: glycogen synthase kinase-3β; PI3K: Phosphoinositide 3-kinase; ROS: reactive oxygen species; TGF-β1: transforming growth factor-β1; ER stress: endoplasmic reticulum stress; (**B**). Autophagy in podocytes. PI3K: Phosphoinositide 3-kinase; AMPK: AMP-activated protein kinase Akt: also stand for PKB (Protein kinase B); ROS: reactive oxygen species; ER stress: endoplasmic reticulum stress; (**C**). Autophagy in proximal tubular cells. IRI: ischemic reperfusion injury; ROS: reactive oxygen species; ER stress: endoplasmic reticulum stress; PI3K: Phosphoinositide 3-kinase; BUN, blood urea nitrogen; sCr, serum creatinine.

After the environmental toxin cadmium exposure, the reactive oxygen species (ROS) increases and activates glycogen synthase kinase-3β (GSK-3β) to trigger autophagy that promotes mesangial cells death [3,18]. Cadmium exposure can also increase autophagy through Ca^2+^ signaling and mitochondrial depolarization pathway and serve as a housekeeping process to protect the kidney and an early biomarker for cadmium toxicity [18] (Figure 2A).

### 2.2. Autophagy in Podocytes

Autophagy is vital in post-mitotic cells, such as neuron cells and podocytes. [19]. Podocytes are highly differentiated epithelial cells located in the visceral site of the Bowman’s capsule and surrounding capillaries of the glomerulus. Podocytes have characteristic foot processes called pedicles that wrap around the capillaries and extend into them to form a filtration slit diaphragm. The podocyte foot processes and slit diaphragm control the selective permeability of the glomerular filtration barrier that filters circulating blood to form urine [20]. Podocytes can also act as glomerular filtration rate regulators through contraction and filtration slit closure [21]. Podocyte injuries are typical hallmarks of acute kidney injury and can cause proteinuria and nutrient loss [19,20]. The degree of podocytes damages parallels the severity of glomerulosclerosis, proteinuria, and kidney injury [22].

Studies in mice suggested that podocytes exhibit a high basal level of autophagy with abundant autophagosomes [3]. Nine-week-old mice with podocyte-specific deletion of Class III PI3K vacuolar protein sorting 34 (Vps34), which helps to maintain autophagic regulation, develop early proteinuria, progressive glomerulosclerosis, and renal failure [3]. The podocyte-specific depletion of the Atg5 gene leads to glomerulopathy in aging mice, whose oxidative and ubiquitinated protein accumulation and podocyte endoplasmic reticulum stress eventually lead to loss of podocytes, increased proteinuria, and glomerulosclerosis [20,22]. Moreover, the inadvertently increased autophagosomes in podocytes can be found in different glomerular diseases such as IgA nephropathy, membranous glomerulonephritis, and focal segmental glomerulosclerosis [20,23].

The autophagy activated by mTOR pathway protects podocytes from apoptosis, foot process effacement, and chronic kidney diseases progression [24]. In podocyte-specific mTor knockout mice, the proteinuria and end-stage renal diseases (ESRD) occur in 3–5 weeks. Their podocytes accumulate with autophagosomes, microtubule-associated protein 1A/1B-light chain 3 (LC3), and damaged mitochondria [25]. The evidence suggest that autophagy could be regulated through the mTOR pathway in podocytes in both rat models and human. The physiologic level of mTOR activity inhibits autophagy and maintains autophagosomes at a basal level to remove damaged organelles, excessive lipids, and long-lived or misfolded proteins in podocytes. The decrease of growth factor signal or exogenous stimulation such as starvation or rapamycin could inhibit mTOR pathway and upregulate autophagy function (Figure 2B). The activation of autophagy as demonstrated by increase LC3 and Lysotracker markers enhanced autophagosome and autophagolysosomes formation [25]. The renal biopsy study demonstrates that podocyte markers such as synaptopodin, podocin, CD2AP, and nephrin were also decreased after being treated with sirolimus in podocytes [26]. Although the mechanism is not completely understood, the evidence indicates that autophagy plays a major role in maintaining podocyte homeostasis and renal function.

### 2.3. Autophagy in Renal Tubular Cells

Autophagy is important in proximal tubular cells. Proximal tubular cells consume much energy during electrolyte reabsorption, which requires high lysosomal activity and mitochondrial turnover [27]. The Atg5 deletion in both proximal and distal tubules results in severe tubular damage and renal dysfunction. However, distal tubules only-specific Atg5 knockout mice show no tubular damage and have normal renal functions [20,28,29]. This indicates that autophagy is critical and integral in proximal tubule function while distal tubular cells rely less on autophagy for their homeostasis due to its function being more passive and less energy dependent [30]. The tubular cells are vulnerable to renal toxic agents, such as cisplatin, cyclosporin, and cadmium [3,31]. Autophagy activation protects renal tubular cells from these renal toxic agents and eliminates the damaged proteins and DNA [3,11]. Selective tubular cell *Atg5* and *Atg7* knockout mice develop more severe tubular cells damages and acute kidney injury (AKI) after ischemia-reperfusion injury [30]. Another study that applied PI3K inhibitor 3-MA to reduced autophagosome sequestration, revealed that it can significantly reduce autophagy function and cause more severe elevation of BUN and serum Cr while rapamycin treatments showed the opposite effect after ischemia-reperfusion injury in vivo and in vitro [8] (Figure 2C). Moreover, constant autophagy activations lead to tubular cells atrophy and promote kidney fibrosis. A delicate balance of the autophagy effects will be required to protect the renal proximal tubular cells from nephrotoxicity drugs and ischemic-reperfusion injury [13].

## 3. Autophagy in Acute Kidney Injury

AKI is a common clinical condition in critical care units. It presents as an abrupt decline of kidney function and imbalance of water, electrolytes, and protein homeostasis [9]. Patients with AKI are associated with higher morbidity and mortality. Major causes of AKI are infections, nephrotoxins, and ischemia-reperfusion injury all inducing inflammation. These diseases will result in direct tubular cell damage, accumulation of oxidative stress (ROS), and endothelium microvasculature dysfunction [9]. Among all, the ischemia-reperfusion injury is considered the most frequent and important etiology that usually causes severe injury to the renal tubular cells [32,33].

### 3.1. The Kidney Ischemia-Reperfusion Injury and Autophagy

The ischemia-reperfusion injury occurs when an organ is exposed to a prolonged duration of blood flow restriction with subsequent restoration of perfusion. The reoxygenation after ischemia will exacerbate the tissue injury and inflammation response [34]. This pathophysiological process is common in many diseases, such as myocardial infarction [35], ischemic stroke, acute kidney injury, trauma, sleep apnea, hypovolemic shock, surgery, and organ dysfunction after transplantation [34]. After ischemia-reperfusion occurs, reactive oxygen species (ROS) in mitochondria will increase and alter cell cycle, damage DNA, and lead to cell dysfunction and death [36]. These ROS and damaged mitochondria are the major upstream cellular signals for the autophagy during renal injury [37,38]. Previous data suggested that ischemia-reperfusion injury to the renal tubular cells results in upregulating the autophagic activity [20,28]. The accumulation of apoptotic cells also activates the autophagy. The activation of autophagy after ischemia is rapid and proceeds to tissue damages or tubular apoptosis [39]. However, newer evidence suggested that prolonged autophagy activations may have adverse effects after ischemic injury in mice. The persistent autophagy activation may trigger renal cell death pathways and exaggerate the kidney damage [9,20]. Although the exact mechanism responsible for the autophagy activation after AKI is still controversial, the autophagy activation after AKI is crucial for the renal protections after AKI.

### 3.2. Autophagy Protects the Renal Cells from Acute Injury

Many studies have proven that the autophagy has renoprotective effects on the proximal tubular cells during AKI [37,39]. Deletion of global Atg5 in mice results in a more vulnerable tubular cell phenotype after exposure to hypoxemia and ROS [27]. Mice with the proximal tubules-specific Atg5 knockout exhibits an accumulation of the damaged organelles and proteins in the proximal tubules and irreversible kidney injury [30,40]. The proximal tubule-specific Atg7 knockout mice also have increased renal injuries [31]. Renal protective role of autophagy has also been shown in cisplatin-induced AKI and sepsis-induced AKI. During the resolution phase of the AKI, modulation of autophagy can promote tubular cell regeneration and repair [8,27]. Moreover, the severity of AKI is associated with the possibility to progress to chronic kidney disease (CKD). Around 15% to 20% of patients with AKI advance to end-stage renal disease [41]. It is important to study the role of autophagy affecting the transition from AKI to CKD.

## 4. Autophagy in Chronic Kidney Disease

The incidence and prevalence of the CKD are increasing in the past two decades [32,42,43]. The CKD is a multifactorial disease with two major causes of CKD being diabetes and hypertension [43]. Other common causes of CKD include glomerulonephritis, polycystic kidney disease, kidney stones, urinary infections, drugs, and nephrotoxins [44]. The increasing prevalence of CKD has become a great burden to the healthcare system worldwide [32,44].

### 4.1. Pathophysiology

Currently, the exact mechanism of CKD is still unknown. The final common pathway of CKD involves glomerulosclerosis, vascular sclerosis, and tubulointerstitial fibrosis. The progression of the CKD involves complex mechanisms, including glomerular hypertension, renin-angiotensin-aldosterone signaling, podocyte homeostasis, dyslipidemia, tubulointerstitial fibrosis, and genetic factors. Recent advances also show that autophagy has an important role in CKD [45,46,47]. CKD patients have elevated oxidative stress and increased ROS production in mitochondria in addition to altered body homeostasis, protein aggregation, and inflammation. Autophagy is essential in keeping the balance of body homeostasis and protein recycling. Autophagy activation is critical in inflammatory responses. Moreover, oxidative stress and ROS are both important regulators of autophagy. Clinical data also show that patients with CKD have altered autophagy responses [48].

### 4.2. Our Previous Research

According to the 2016 guideline for autophagy monitoring, there is no absolute criteria that are applicable in every clinical or experimental context for determining autophagic status. Although some autophagy markers have been used to estimate the autophagic activity in patients, such as increase synthesis or lipidation of LC3 and increase autophagosomes formation [49]. However, it is difficult to measure the exact autophagy flux in clinical settings.

In 2013, we have designed a method to measure the autophagic function in leukocytes from patients. LC3 proteins are involved in phagophore formation and characterized as autophagosome markers. A cytosolic form of LC3 (LC3-I) is conjugated to phosphatidylethanolamine to form LC3-II, which usually reflects the formation of autophagosomes. Previous studies showed that the LC3-I level is very stable during starvation and that the LC3-II level is reflective of changes in the autophagic function and flow. We, therefore, postulated that LC3-I can serve as an ideal control in human leukocytes. The ratio of the 14 kDa LC3-II versus the 16 kDa LC3-I (LC3-II/LC3-I) in leukocytes can serve as an indicator of autophagy flux. The ratio of LC3-II/LC3-I after fasting for 12 h (LC3-II/LC3-I-AC) versus LC3-II/LC3-I 2 h after breakfast (LC3-II/LC3-I-PC) in the same subject can be calculated as γLC3 and regarded as an indicator of autophagy flux or activation. We enrolled 60 patients diagnosed with stages 4–5 CKD (30 with hemodialysis and 30 without hemodialysis), and 30 healthy volunteers as the control group who were sex- and age-matched. In the CKD with hemodialysis group, the blood sample was collected one day after hemodialysis. Using γLC3 as the marker, we have measured the autophagy flux in CKD patients. The isolated LC3-I or LC3-II after fasting or feeding showed no significant associations with healthy subjects and CKD patients with or without hemodialysis. Overnight fasting increased autophagy flux and γLC3 in healthy subjects, which were nearly absent in CKD patients. Moreover, hemodialysis could not correct the autophagy flux deficiency in CKD patients. The *Atg5* and *Beclin-1* transcript levels also increased after starvation in the healthy group, while there were no significant changes in the CKD group. Our data thus provided the direct evidence supporting that CKD patients have impaired autophagy activation and could not be reversed by hemodialysis. The γLC3 was a better autophagic activity indicator then the isolated LC3-I or LC3-II [48].

We also highlighted the relationship between cardiovascular diseases in CKD patients using echocardiography to measure the cardiac functions and structures. The γLC3 was negatively associated with left atrial sizes; changes of the *Atg5* transcript were negatively associated with LVEDD, and; changes of the *Beclin-1* transcript were negatively associated with diastolic mitral inflow E- and A-wave values. The different autophagy markers are associated with different echocardiographic parameters [48]. Because the increased LA size correlates with the increased incidence of atrial fibrillation, stroke, acute myocardial infarction, and congestive heart failure [50], our observation suggested a close relationship of autophagy and CKD-related cardiovascular diseases. The exact mechanism of how CKD has autophagy deficiency is still unclear and will require further studies.

### 4.3. Diabetic Nephropathy

Diabetic nephropathy is a major cause of CKD and end-stage renal disease worldwide [51,52]. Olivia Lenoir and colleagues have reported that high glucose concentration environs activated autophagy in podocytes and protected the podocytes from hyperglycemia-related apoptosis [47]. Deficiency of autophagy activation by knockout Atg5 in diabetic mice resulted in more severe proteinuria and impaired renal function [52,53]. Impaired autophagy in the kidney also resulted in podocyte loss and massive proteinuria in diabetic nephropathy [54]. Decreased mTORC1 activation in diabetic mice could stimulate autophagy and decrease glomerulosclerosis, proteinuria, and podocyte loss to slow down progression to diabetic nephropathy [55].

However, there were some studies suggesting the opposite effect of high glucose on podocyte. Through CASP3 activation, high glucose lead to podocyte apoptosis [56]. With the high glucose stimulation, human podocytes exhibited a dramatically reduced LC3-II and Beclin-1 and decreased autophagy activation [57]. The controversies are still unsolved, and more evidence is needed for making the conclusion.

Currently, only a few studies have described the relationship between autophagic markers and podocyte-specific proteins. The podocin protein is a key protein of the slit diaphragm of podocytes. In patients with diabetic nephropathy and severe proteinuria, their kidney biopsy samples express podocin with a granular and irregularly scattered pattern under immunofluorescent study, whereas intense accumulation of p62 proteins is presented in glomeruli. In the 50-week-old diabetic nephropathy rats model with massive proteinuria, the podocytes showed a reduction in podocin-positive areas, p62 accumulation, a decrease of LC3-II, and alteration of foot processes. These suggest that the insufficient autophagic function could cause podocytes injury in diabetic nephropathy with severe proteinuria [58].

Autophagy also protects mesangial cells from undergoing apoptosis in diabetic nephropathy after induced by TGF-β1 via TAK1 and PI3K–AKT-dependent pathways [55]. In proximal tubular cells, autophagy activation is reduced by hyperglycemia while p62 is increased in both type 1 and type 2 diabetes animal models [59,60]. Studies indicated that diabetic nephropathy is associated with decreased autophagy activity and increased apoptosis [54,55,61]. These evidence support that autophagy can be a therapeutic target for diabetic nephropathy [60].

### 4.4. Autoimmune Kidney Disease

Autophagy may regulate autoimmune responses by modulating innate immunity and lymphocyte homeostasis [62]. Several studies have manifested the relationship between autoimmune diseases and autophagy in both animal models and human studies. Autophagy helps to understand the mechanism of autoimmune diseases and open the possible therapeutic strategies in systemic lupus erythematosus (SLE), Sjögren’s syndrome, Crohn’s disease, rheumatoid arthritis, multiple sclerosis, and type 1 diabetes mellitus. [63,64]. The Lupus nephritis is the most common of all and manifested as severe complications of the SLE. Severe lupus nephritis can lead to end-stage renal disease and is an important predictor of mortality in SLE patients [65]. The lupus nephritis results from complement activation, autoantibody formation, immune complexes formation, and dysfunctional adaptive immune responses. The autophagy interacts with these processes and preserves renal function [66]. Some SLE patients have activated autophagic genes, such as *Atg5* and *Atg7*. They also have increased autophagic vacuoles in B cells, T cells, and macrophage in peripheral blood mononuclear cells [64,67]. Current evidence suggest that lupus nephritis may be associated with renal Epstein–Barr virus infection, which can induce autophagy in B cells in a dose-dependent manner [68,69]. In the mice lupus nephritis model, podocytes exhibit autophagy activation that protects renal function from deterioration. The mice podocytes from lupus nephritis have increased autophagosomes, increased LC3-II/LC3-I ratios, and decreased p62 [70]. The aggregated lupus autoantibodies can assist injured podocytes being degraded by autophagy activation [71]. Several drugs given to SLE patients are mTOR inhibitors which can induce the autophagy activity, suggesting autoimmune diseases are related to autophagy dysfunction and therefore give rise to future possible therapeutic intervention options [64].

### 4.5. Infection

Severe systemic infection and sepsis induce a cytokine storm and influence multiple tissues and organs including the kidney. Autophagy is up-regulated early after sepsis and protects organs from pathogen by modulating the immune systems and regulating macrophage, dendritic cells, B cells, CD4^+^, and CD8^+^ T cells functions. [63,72]. Autophagy has been suggested to protect kidneys against septic kidney injury [41,66,73]. In the cecal ligation and puncture mice model, the sepsis induced by the peritoneal infections activates the autophagy. The activated autophagy protects the kidney function by decreasing circulating cytokines and endothelial activation [41]. In a sepsis rat model, the decline of autophagy response is associated with the development of kidney injury. Knockdown of *Atg7* decreases tumor necrosis factor α-related proximal tubular cell death, which can be reversed with rapamycin [73]. Autophagy harbors the capacity to both pro- and anti-inflammatory responses to suppress sepsis-induced kidney injury through regulation of infection and through targeting inflammasome and type I interferon responses [66]. These observations suggest that autophagy may be a therapeutic target to protect kidney injury from sepsis.

### 4.6. Renal Tubulointerstitial Diseases and Ureter Obstruction

On 2010, Li and colleagues demonstrated that autophagy was significantly activated in a unilateral ureteral obstruction mice model. The conversion of LC3-I to LC3-II, activation of Beclin-1, and accumulation of autophagosomes with massive autophagic vesicles were observed in atrophic tubules with tubulointerstitial injury [74]. Mice with LC3B knockout exhibited a deficit in autophagy activation and severe tubulointerstitial fibrosis after ureter obstruction [75]. The development of tubular atrophy and nephron loss correlated with autophagy in a time-dependent manner [13]. However, persistent activation of autophagy in kidney tubular cells would promote renal interstitial fibrosis through fibroblast growth factor 2 [76]. These studies suggested that the balance of autophagy activation is important in regulating the tubulointerstitial function and renal fibrosis in obstructive kidney disease [27].

### 4.7. Toxic Effects of Drugs

Nephrotoxicity of many therapeutic medications can cause acute kidney injuries and worsening renal function in patients with CKD. The non-steroidal anti-inflammatory drugs, iodinated contrast medium, and cisplatin are important drugs causing nephrotoxicity related acute kidney injuries. Recently, in vitro studies have shown that iodinated contrast leads to enhancement of mitophagy, and may protect kidneys from iodinated contrast related renal tubular epithelial injury. The mitophagy is one type of the autophagy that can selectively remove the damaged mitochondria [77]. Cisplatin-induced AKI involves multiple mechanisms, including proximal tubular injury, oxidative stress, inflammation, and vascular injury. The injury is predominantly acute tubular necrosis in the proximal tubules [78,79,80]. Previous studies suggest that the autophagic responses to cisplatin treatment may protect many types of cancer cells and result in cisplatin resistance [78]. Cisplatin also activated autophagy in the proximal tubules with massive autophagosome formation and LC3-II accumulation for protection purpose [79,81]. Also, in proximal tubule-specific Atg5-knockout mice, cisplatin prompted more severe DNA damage and p53 activation, as well as accumulated more protein aggregates in proximal tubules [79,82]. Furthermore, rapamycin, an mTOR inhibitor, could treat the cisplatin-induced AKI in mice to improve renal function [78,79]. Oral anti-diabetic agents, metformin, also activated the autophagy and protected against cisplatin-induced tubular injury by activating autophagy cascades and slowing down the apoptosis of tubular cells [83]. These data indicated that autophagy can protect renal tubule injury against cisplatin [29].

**Figure 3:**
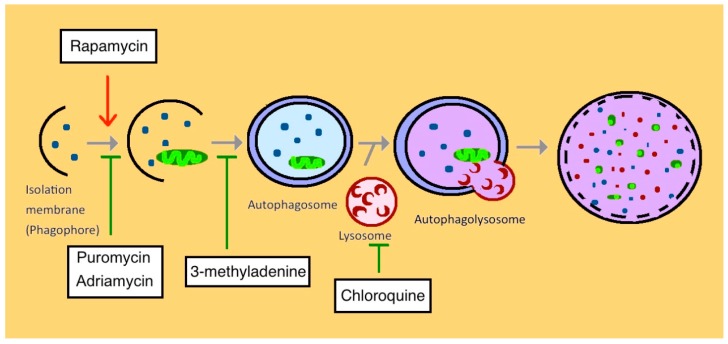
Overview of the different medications that regulate autophagy in different steps, including Rapamycin, Adriamycin, Puromycin, 3-methyladenine and Chloroquine.

The Adriamycin- and Puromycin aminonucleoside-induced podocyte apoptosis is widely used for studying the pathophysiology of glomerular diseases in vitro and in vivo. The activation of autophagy with rapamycin could suppress the Adriamycin-induced apoptosis while inhibiting autophagy with chloroquine enhanced apoptosis. The podocyte-specific Atg7 knockout mouse model described the aggravated podocyte injury, glomerulopathy, and proteinuria after adriamycin treatment [84]. The upregulation of LC3-positive autophagosomes protects puromycin aminonucleoside-induced nephrosis in rats in vivo and immortalized mouse podocytes in vitro [85]. Puromycin aminonucleoside reduces the autophagy in human podocytes with the activation of mTORC1. When inhibiting autophagy with the 3-methyladenine or chloroquine, the podocyte apoptosis increased significantly along with the elevation of active caspase-3 [86]. The rapamycin activated autophagy led to decreased proteinuria and less severe foot-process effacement [87] (Figure 3). Collectively, this evidence supports autophagy may be an early adaptive cytoprotective mechanism for podocytes under Adriamycin and puromycin aminonucleoside-induced apoptosis intervention.

Although evidence has explicated the relationship between toxin/drug and the autophagy in the kidney, future studies are still necessary to investigate the mechanism of autophagy in many other nephrotoxic agents.

### 4.8. Cystic Disease (Polycystic Kidney Disease)

Autosomal dominant polycystic kidney disease (ADPKD) is one of the most prevalent inherited renal cyst diseases and frequently leads to end-stage renal disease. The main cause of this disease is gene mutation. Approximately 85% of the mutations in ADPKD occur in the *pkd1* gene encoding for polycystin-1, and 10–15% in its interaction partner *pkd2*, encoding for polycystin-2. These ciliary proteins are involved in cellular repair and growth mechanisms [88].

Both in vitro and in vivo studies have shown that multiple autophagic molecular parameters and signaling pathways are involved in ADPKD, including mTOR, cyclic adenosine monophosphate, and several growth factors [89]. The current evidence suggests that autophagy processes may relate to the cystic formation and size growth by activating the mTOR signaling pathway [89,90]. ADPKD is a cilia-related disease and autophagy activation is essential in ciliogenesis [12]. Studies revealed that autophagy regulates cilia length by modulating protein synthesis and degradation. Through modulating autophagy activation, the primary cilium controls epithelial cell volume in a fluid flow dependent manner [91]. The ADPKD exhibiting autophagosome increases with LC3-II and Beclin-1 overexpression in tubular cyst-lining cells, suggesting autophagic flux dysregulation [1]. In a mice model with mutated PKD1 and patients with ADPKD, autophagy activation is impaired [92]. Polycystin-1 negatively regulates polycystin-2 via the autophagosomes-dependent pathway. Failure of pathogenic polycystin-1 mutants to induce this function may lead to ADPKD [93]. All this evidence suggests that the ADPKD may be present with dysfunctional autophagy [1,94]. Currently, the regulation of autophagy in ADPKD is incompletely understood. Autophagy may be one of the therapeutic targets in the future.

### 4.9. Autophagy in Aging-Associated CKD

Aging is associated with a deterioration and imbalance of the general homeostasis ability, which results in loss of the compensation mechanism in the kidneys. In the elderly population, the prevalence of non-dialysis CKD is markedly higher. The normal aging kidneys exhibit decreases in renal mass, increases in parenchymal tissue fibrosis and fat deposition, and accumulation of glomerular sclerosis [95,96]. Development of CKD is a major risk factor of ESRD in the elderly population. The mortality among the age of 75 with CKD is double compared to the healthy population [97,98].

The podocyte-specific *Atg5* knockout mice develop glomerulopathy gradually during aging. The podocytes without *Atg5* have decreased organelles turnover with the accumulation of ubiquitinated and oxidative protein. The deficiency of autophagy and proteasome pathways leads to proteinuria, loss of podocytes, and development of glomerulosclerosis in aging mice [22]. In aging mice, podocyte-specific autophagy-deficient mice have mild forms of glomerulosclerosis compared with tubular cell-specific autophagy-deficient mice [66]. Yamamoto and colleagues demonstrated that proximal tubule-specific deletion of Atg5 in mice resulted in significant deteriorations of kidney function and fibrosis at 24 months of age [99]. These results indicate that tubular cells play an important role in aging-related autophagy regulation of kidney function. The inability of older mice to recover from AKI has been attributed to an age-dependent loss of autophagy resulting in CKD [41]. These results suggest that autophagy is vital for maintaining kidney tissue homeostasis and aging-associated injury [3].

Caloric restriction has been shown to strongly induce autophagy in kidneys and slow down the process of interstitial fibrosis and tubular atrophy [100]. Moreover, caloric restriction prolongs lifespan in animals and decreases the age-related mitochondrial oxidative damage and the kidney tissue injury by enhancing autophagy [101,102]. Caloric restriction in AKI with rats of different ages suggests that the caloric restriction can significantly increase autophagy activation by increasing LC3-I/LC3-II ratio and improving renal function. The nephroprotection effects gradually decline with the age due to deteriorations of the autophagic system [103]. The mTOR pathway acts as a two-way regulator during starvation and caloric restriction in rat. At first, the mTOR inhibition activates autophagy to form autophagolysosome and breakdown the cellular content for regeneration to further protect the kidney function. However, prolonged starvation will reactivate the mTOR pathway and inhibit autophagy by negative feedback and accumulation of autophagolysosome degradation content. [104]. Therefore, a balanced caloric restriction strategy taking into consideration of the autophagy activation is one of the therapeutic options to delay the progression of CKD among elderly patients [100].

## 5. Autophagy in Dialysis and Renal Transplantation

The end-stage renal disease is the most severe outcome of CKD and can only be treated with dialysis or renal transplantation. Patients with end-stage renal disease have increased risks of cardiovascular disease, cerebrovascular accident, infection, and cognitive impairment [44].

Until recently, very few studies have investigated the relationship between dialysis and autophagy. Our previous study indicated that autophagy flux could not be rescued by hemodialysis in patients with end-stage renal disease, suggesting that hemodialysis has no role of modulating autophagy [48]. When it comes to peritoneal dialysis (PD), one study suggests that autophagy stimulates the fibrosis and apoptosis in peritoneal mesothelium cells due to long-term exposure to the high-glucose peritoneal dialysis solution. The activation of Beclin 1-dependent autophagy results in decreases of the viability of peritoneal mesothelium cells and finally PD failure [105]. Another study, however, suggests that the PD solution promotes autophagosome formation and decreases cells death to maintain the PD function [106]. Therefore, future studies are required to understand the exact relationship of dialysis and autophagy regulation.

Renal transplantation is considered as the definitive treatment to ESRD. Currently, some evidence supports the connections between autophagy and renal transplantation. The mTOR inhibitor is frequently used in renal transplantation as an immunosuppressant, including rapamycin, sirolimus, and everolimus. Rapamycin inhibits T cell proliferation and activates autophagy to maintain the renal homeostasis [107,108]. Cyclosporine is another immunosuppressive drug commonly used in renal transplantation patients [109]. Cyclosporine induces autophagy in primary cultured renal tubular cells. After treatment of cyclosporine in mice, the kidney proximal tubule cells exhibit an increase in numbers and sizes of autophagosomes and autophagy flow [110]. Deficiency in autophagy function will lead to an exacerbation of cyclosporine-related endoplasmic reticulum stress and renal injury [110,111]. Another indirect clue is that a kidney from elderly donors is now considered to have a higher risk of post-transplant ischemic injury, which may be due to the decline of autophagy activation with aging [107,112].

## 6. Therapeutic Consideration and Conclusion

Autophagy defects can occur at different stages of the pathway in CKD, and this may influence treatment strategies. Early studies examine the rapamycin through upregulating autophagy to enable the clearance of intracytoplasmic aggregation-prone proteins. Rapamycin is currently the only drug that could be used to target autophagy in CKD treatments. However, the long-term side effects of rapamycin should be taken into consideration [7]. Some studies have used the rapamycin under intermittent dosing protocols in mice that could create pulsatile upregulation of autophagosome formations. This gives us a possible therapeutic regimen to reduce rapamycin side effects in patients.

### 6.1. Future Therapeutic Considerations

Several candidate drugs targeting autophagy have been shown to inhibit or activate the autophagy function. However, many of them are still under animal trials or early phase clinical trials and are not ready to be used clinically. Some drugs that are used clinically for other diseases also are found to have the potential to regulate autophagy through different autophagic pathways. The psychotropic drug lithium, carbamazepine, and valproic acid can activate autophagy through the phosphatidylinositol signaling pathway. The clonidine and rilmenidine act as the imidazoline receptor agonists to enhance autophagy. Verapamil targets L-type Ca^2+^ channels to modulate autophagic flux. Metformin upregulates AMPK signaling and induces autophagy function. In contrary, chloroquine is a lysosomotropic agent that inhibits autophagosome-lysosome fusion and lead to autophagy downregulation [113]. Ultimately, whether autophagy represents a useful target in CKD prevention or treatment will need to be addressed by conducting clinical trials in patients in the future.

### 6.2. Conclusions

**Figure 4:**
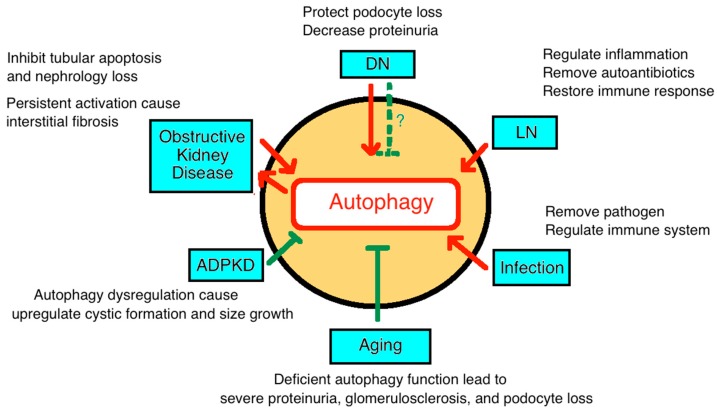
Diagram depicting the roles of autophagy in various chronic kidney disease. DN: diabetic nephropathy; LN: lupus nephropathy; APCKD: adult polycystic kidney disease.

We summarize the autophagy pathophysiology in chronic kidney disease (Figure 4). The exposure of normal kidneys to selective stresses, such as ischemic-reperfusion injury, toxin, and sepsis, will result in acute kidney injury, ROS accumulation, and autophagy activation. The protective mechanisms of autophagy take place in podocytes, mesangial cells, and tubular cells, help repair and regenerate the damaged kidneys. After several episodes of stresses, the balancing by the autophagic repair mechanism cannot keep up and CKD ensues. Different CKDs also modulate autophagy in diverse pathways and help to slow down the progression to ESRD as in Table 1.

At the turning point of autophagy research in the early 1990s with the identification of the autophagy-related gene, many researchers have devoted their efforts to finding the potential therapeutic use of autophagy in human diseases. The relationships between autophagy and kidney diseases are well-founded, but there is still no clinically useable agent targeting the autophagic pathway for patients with CKD or AKI. The complex nature of CKD or AKI further complicates the development of therapeutic advancement. The evidence from mice or cells have provided us with comprehensive mechanistic insights into the etiology and pathophysiology of the disease. Since autophagic machinery has a vital role in controlling immunity, it will be crucial for clinicians to monitor the occurrence of infection and autoimmune diseases with autophagy inducers or inhibitors [104]. Besides, inhibition or activation of the autophagy will impact multiple aspects of organism physiology with various effects [114,115,116]. Therefore, it is necessary to take these diverse interactions into consideration when developing autophagy-targeting drugs. In the end, medications developed by tackling these mechanistic pathways still require prospective and randomized trials to verify their therapeutic potentials and adverse events when administered in humans.

## Figures and Tables

**Table 1:** Overview of the autophagy in chronic kidney diseases (CKD).

CKD Categories		Reference
Diabetes Nephropathy (DN)	**Increase**	
	High glucose environment activates autophagy in podocytes	[47]
	*Atg5* knockout mice with DM result in more severe proteinuria and renal failure	[52,53]
	Reduced mTORC1 activation stimulates autophagy and protects DN progression	[55]
	DN activates TGF-β1 via TAK1 and PI3K–AKT-dependent pathways and protects mesangial cells from apoptosis	[55]
	**Decrease**	
	High glucose activates CASP3 and leads to podocyte apoptosis	[56]
	High glucose reduces LC3-II and Beclin-1 in podocytes and decreases autophagy activity	[57]
	High glucose reduces autophagy activity in proximal tubular cells	[59,60]
	DN decreases autophagy activity and increase apoptosis	[54,55,61]
Lupus nephritis (LN)	**Increase**	
	SLE activates autophagic genes (*Atg5* and *Atg7*) and increases autophagic vacuoles in B cells, T cells and macrophage in peripheral blood mononuclear cells	[64,67]
	EBV related LN induces autophagy in B cells in a dose-dependent manner	[68,69]
	Podocytes increase autophagosomes, increase LC3-II/LC3-I ratios, and decrease p62 to protect renal function	[70]
	mTOR inhibitor improves LN by inducing autophagy activity	[64]
Infection	**Increase**	
	Early autophagy up-regulation after infection modulates the immune system and regulates immune cells function including macrophage, dendritic cells, B cells and CD4^+^, and CD8^+^ T cells.	[63,72]
	Cecal ligation and puncture mice model activates autophagy to protect renal function	[41]
	*Atg7* knockout mice increase tumor necrosis factor-α promote tubular cells death. Rapamycin may reverse the effect.	[73]
	Autophagy regulates infection through targeting inflammasome and type I interferon responses	[66]
**Tubulointerstitial injury**	**Protect**	
	UUO increases conversion of LC3-I to LC3-II, activation of Beclin-1, and accumulation of autophagosomes	[74]
	LC3B knockout mice have more severe tubulointerstitial injury	[75]
	Tubular atrophy and nephron loss correlate with autophagy in a time-dependent manner	[13]
	**Damage**	
	Persistent activation of autophagy promotes interstitial fibrosis through fibroblast growth factor 2	[75]
**Toxic/Drugs**	**Iodinated contrast**	
	Enhance mitophagy protects kidneys from tubular epithelial injury	[77]
	**Cisplatin**	
	Autophagic activation protects some cancer cells and results in cisplatin resistance	[78]
	Cisplatin activates autophagy in the proximal tubules with autophagosome formation and LC3-II accumulation	[79,81]
	*Atg5* deficiency results in more severe tubular damage and can be reversed by rapamycin	[78,79,82]
	Metformin increases autophagy and protects renal from cisplatin-induced tubular injury	[83]
	Adriamycin/Puromycin aminonucleoside	
	Adriamycin and Puromycin aminonucleoside activate mTOR pathway to inhibit autophagy causing podocyte apoptosis	[84,85]
**Autosomal dominant polycystic kidney disease** **(ADPKD)**	**Dysregulation**	
	Autophagy is related to the cystic formation and size growth by activating the mTOR signaling pathway. Autophagy regulates cilia length by modulating protein synthesis and degradation	[89,90,91]
	Increased autophagosome formation and LC3-II/Beclin-1 overexpression in tubular cyst-lining cells;ADPKD may present with dysfunctional autophagy	[1,94]
**Aging-Associated CKD**	**Deficiency**	
	Autophagy deficiency leads to proteinuria, loss of podocytes, and development of glomerulosclerosis in aging mice	[22]
	Proximal tubule-specific *Atg5* deletion mice result in significant deteriorations of kidney function and fibrosis at 24 months of age	[99]
	Inability of older mice to recover from AKI in an age-dependent manner	[41]
	Caloric restriction prolongs lifespan and decreases kidney tissue injury by enhancing autophagy	[101,102]
